# Nicotine Causes Nephrotoxicity through the Induction of NLRP6 Inflammasome and Alpha7 Nicotinic Acetylcholine Receptor

**DOI:** 10.3390/toxics8040092

**Published:** 2020-10-26

**Authors:** Cai-Mei Zheng, Yu-Hsuan Lee, I-Jen Chiu, Yu-Jhe Chiu, Li-Chin Sung, Yung-Ho Hsu, Hui-Wen Chiu

**Affiliations:** 1 Division of Nephrology, Department of Internal Medicine, School of Medicine, College of Medicine, Taipei Medical University, Taipei 11031, Taiwan; 11044@s.tmu.edu.tw; 2 Division of Nephrology, Department of Internal Medicine, Shuang Ho Hospital, Taipei Medical University, New Taipei City 23561, Taiwan; stirbar2000@yahoo.com.tw (I.-J.C.); chiuyj2002@gmail.com (Y.-J.C.); 3 TMU Research Center of Urology and Kidney, Taipei Medical University, Taipei 11031, Taiwan; 4 Department of Cosmeceutics, China Medical University, Taichung 40604, Taiwan; bmm175@hotmail.com; 5 Graduate Institute of Clinical Medicine, College of Medicine, Taipei Medical University, Taipei 11031, Taiwan; 6 Division of Cardiology, Department of Internal Medicine, School of Medicine, College of Medicine, Taipei Medical University, Taipei 11031, Taiwan; 10204@s.tmu.edu.tw; 7 Division of Cardiology, Department of Internal Medicine, Shuang Ho Hospital, Taipei Medical University, New Taipei City 23561, Taiwan; 8 Taipei Heart Institute, Taipei Medical University, Taipei 11031, Taiwan

**Keywords:** nicotine, alpha7 nicotinic acetylcholine receptor, NLRP6 inflammasome, autophagy, endoplasmic reticulum stress

## Abstract

Current cigarette smoking is associated with chronic kidney disease (CKD) or death from end-stage renal disease (ESRD). Mainstream cigarette smoke includes over 4000 compounds. Among the compounds present in tobacco smoke, nicotine is one of a large number of biologically stable and active compounds present in tobacco. However, the mechanisms by which nicotine exacerbates kidney disease progression have not been identified. It is known that the inflammasomes constitute an important innate immune pathway and contribute to the pathophysiology of diverse kidney diseases. The relationship between inflammasomes and nicotine-induced kidney damage still remains unclear. In the present study, we studied the mechanisms of nicotine-induced nephrotoxicity. We found that nicotine decreased cell viability and induced reactive oxygen species (ROS) generation in human kidney cells. Furthermore, nicotine significantly increased the expression of the alpha7 nicotinic acetylcholine receptor (α7nAChR). Nicotine activated the NLRP6 inflammasome and induced endoplasmic reticulum (ER) stress. Nicotine caused mild apoptosis and necrosis but triggered significant autophagy in human kidney cells. In addition, nicotine induced the NLRP6 inflammasome and autophagy via α7nAChR. In an animal model, the histological analysis in kidney showed evident changes and injury. The results indicated that α7nAChR, IRE1α, LC3 and NLRP6 expression in kidney sections was markedly increased in the nicotine groups. These findings suggest that nicotine causes kidney damage by modulating α7nAChR, NLRP6 inflammasome, ER stress and autophagy.

## 1. Introduction

Cigarette smoking is reported to be a common risk factor for various diseases including kidney disease and kidney cancer [1,2,3]. Nicotine is a major component of cigarette smoke and is responsible for the addictive effects of cigarette smoking [4]. Nicotine may affect some biological process such as cell-mediated immunity, apoptosis and angiogenesis by binding to the nicotinic acetylcholine receptors (nAChRs) [5]. Previous studies have demonstrated that nicotine plays a central role in smoking-mediated renal dysfunction [6]. Nicotine caused apoptosis by inducing reactive oxygen species (ROS) generation and cell cycle arrest, and by activating the MAPK and NF-κB signaling pathways in human renal tubular epithelial cells [7]. Alpha7 nAChR (α7nAChR) is a member of the nAChR family and is a cationic ligand-gated ion-channel [8]. Some reports have suggested that α7nAChR mediates anti-inflammatory effects through cholinergic modulation [9,10]. The accumulated evidence has revealed that the activation of α7nAChR ameliorates myocardial ischemia/reperfusion injury [11]. However, the underlying mechanism of α7nAChR in kidney cells is still unclear.

Inflammasomes are constituents of an important innate immune pathway that regulate caspase-dependent inflammation and cell death [12]. Inflammasome activation involves the formation and oligomerization of a protein complex including an adaptor protein, a nucleotide oligomerization domain (NOD)-like receptor (NLR) and pro-caspase-1 [13]. The canonical inflammasomes are activated by danger signals, pattern recognition receptors or cellular events. Non-canonical inflammasomes can be induced by toxins, intracellular lipopolysaccharides and several signaling pathways [12]. Inflammasome activation leads to cleavage and activation of caspase-1, as well as the secretion of interleukin (IL)-1β and IL-18 [14]. Inflammasomes have many subtypes. The most studied subtypes of inflammasomes are NLRP3 (NOD-like receptor family, pyrin domain-containing 3) and NLRP6. The data accumulated by us and others have indicated that the canonical NLRP3-ASC-caspase-1 axis contributes to the pathophysiology of some kidney diseases [15,16]. Wu et al. indicated that nicotine promoted atherosclerosis via the production of ROS and activation of NLRP3 [17]. NLRP6 is the first member of the NLR family to inhibit innate immune response-related signaling pathways [18]. However, the relationship between NLRP6 and nicotine-induced kidney damage still remains unclear.

Autophagy is a lysosome-mediated cellular process that degrades protein aggregates, damaged organelles and other macromolecules in the cytoplasm. Autophagy can regulate cell survival and death under normal physiological and pathological conditions [19]. It is well known that autophagy has an important role in acute and chronic kidney injury [20,21]. Recent evidence has shown that autophagy inhibits tubulointerstitial fibrosis by restraining smad4-dependent transforming growth factor (TGF)-β and the NLRP3 inflammasome [15]. Nicotine can induce autophagy and promote atherosclerosis via the nAChRs/ROS/NF-κB signaling pathway in vascular smooth muscle cells [22]. Previous studies have demonstrated that the endoplasmic reticulum (ER) stress response can induce autophagy that reduces ER stress by destroying the damaged organelles and unfolded/misfolded proteins [23]. Furthermore, ER stress can activate the unfolded protein response (UPR). The UPR includes three major pathways: pancreatic eukaryotic translation initiation factor 2α (eIF2α) kinase (PERK), inositol-requiring protein 1 (IRE1) and activating transcription factor 6 (ATF6) [24]. The accumulated evidence indicates that ER stress triggers autophagy through the UPR [25,26]. In addition, there are increasing findings that autophagy can inhibit inflammasome activation [27,28]. Saitoh et al. indicated that loss of autophagy-derived related 16-like 1 (Atg16L1), which is a protein essential for the initiation of autophagy, increased caspase-1 activation and secretion of IL-18 and IL-1β in macrophages. Similarly, treatment with an inhibitor of autophagy 3-methyladenine (3-MA) enhances inflammasome activation [28]. The accumulated evidence has revealed that autophagy negatively regulates inflammasome activation in several ways, including removal of endogenous inflammasome activators or inflammasomes and their downstream cytokines [13]. In the present study, we examined the effect of nicotine on the NLRP6 inflammasome, ER stress, autophagy and apoptosis in kidney cells. In addition, we evaluated the relationship between α7nAChR, autophagy and NLRP6 inflammasome in nicotine-induced nephrotoxicity.

## 2. Materials and Methods

### 2.1. Cell Culture and Nicotine Treatment

The HK-2 cells (human kidney proximal tubular epithelial cell line) (ATCC: CRL-2190) and NRK-52E cells (rat renal tubular cell line) (ATCC: CRL-1571) were acquired from the American Type Culture Collection. The HK-2 cells were maintained in keratinocyte serum-free medium with 5 ng/mL recombinant epidermal growth factor and 40 μg/mL bovine pituitary extract (Gibco BRL, Grand Island, NY, USA) at 37 °C and 5% CO_2_. The NRK-52E cells were cultured in Dulbecco’s modified Eagle’s medium supplemented with an antibiotic/antifungal solution and 10% fetal bovine serum at 37 °C and 5% CO_2_. The two cell lines were used between the 20th and 30th passages. For exposure to nicotine (Sigma Chemical Co., St. Louis, MO, USA), fresh 40 mM solutions were prepared and added to the culture medium and mixed gently.

### 2.2. SRB Cell Viability Assay

The sulforhodamine B (SRB) assay is used to analyze cell viability. After incubation for 24 h with various concentrations of nicotine, the cells were washed with phosphate-buffered saline (PBS) and fixed with 10% trichloroacetic acid (TCA) for at least 1 h or overnight. The TCA solution was removed, and the cells were washed. SRB solution (0.1%) was added and incubated for 1 h. Then, 1% acetic acid was added to the cultures, and the cells were oven dried at 60 °C for 20 min. Finally, the cells were dissolved in 20 mM Tris base solution for 30 min, and the optical density was determined at 562 nm in an ELISA reader.

### 2.3. Measurement of Intracellular Reactive Oxygen Species (ROS) Level

The ROS detection assay kit purchased from Biovision Inc. (Mountain View, CA, USA) was used according to the manufacturer’s protocol. Briefly, the cells were seeded in a 96-well plate and adhered overnight. The media were then removed and the adherent cells were washed in ROS Assay Buffer. The ROS Label solution was diluted in ROS Assay Buffer to 1 X and added to the cells in each well. Finally, the fluorescence at Ex/Em = 495/529 nm in each well of the plate was measured.

### 2.4. Western Blotting

Total protein was extracted from cell lysates by collecting cells. The proteins isolated from the cells were loaded at 30 μg/lane to a sodium dodecyl sulfate (SDS) gel. The gel was subjected to electrophoresis, blotted and probed using antibodies, and the targets were detected using a chemiluminescence detection system (Thermo Fisher Scientific, Waltham, MA, USA). Anti-eIF2α (dilution 1:1000), anti-p-eIF2α (dilution 1:1000), anti-IRE1α (dilution 1:1000) and anti-LC3 (dilution 1:1000) antibodies were obtained from Cell Signaling Technology (Ipswich, MA, USA); anti-α7nAChR (dilution 1:1000), anti-ATF6 (dilution 1:1000), anti-caspase 1 (dilution 1:1000) and anti-GAPDH (dilution 1:10,000) antibodies were obtained from Proteintech Group (Chicago, IL, USA); anti-NLRP6 antibody (dilution 1:1000) was obtained from Abgent (San Diego, CA, USA); and anti-ASC antibody (dilution 1:1000) was obtained from Adipogen (San Diego, CA, USA). The densities of the bands were quantified with a computer densitometer (AlphaImager™ 2200 System Alpha Innotech Corporation, San Leandro, CA, USA).

### 2.5. Detection of IL-1β by ELISA

The supernatant of HK-2 cells was collected to detect IL-1β using ELISA (eBioscience, San Diego, CA, USA) according to the manufacturer’s protocol. The optical density of the peroxidase product was analyzed using an ELISA reader (Emax, Molecular Devices, Sunnyvale, CA, USA) at 450 nm. Based on the standard curve, the concentrations of IL-1β in each sample were determined.

### 2.6. Annexin V and Propidium Iodide (PI)

Apoptosis and necrosis were evaluated with an apoptosis detection kit that utilized FITC Annexin V with PI according to the manufacturer’s instructions (BioLegend, San Diego, CA, USA). The cells were collected and washed with PBS. Then, the cells were stained with PI and Annexin V. Finally, the cells were evaluated using a flow cytometer (Becton Dickinson, San Jose, CA, USA). The apoptotic and necrotic cells are presented as percentages of the total cell number.

### 2.7. Immunofluorescence Assay

The cells were seeded on coverslips. After nicotine treatment, the cells were fixed in paraformaldehyde (4%) and blocked with bovine serum albumin (1%) for 30 min. Then, the cells were incubated with an anti-LC3 antibody (MBL, Japan) in blocking solution for 1 h. After washing, the cells were stained with goat anti-rabbit DyLight™ 488 (Jackson ImmunoResearch Laboratories, PA, USA) antibodies in blocking solution for 1 h and 4′,6-diamidino-2-phenylindole (DAPI) (Invitrogen, Carlsbad, CA, USA). Finally, the cells were washed in PBS and images were taken with a fluorescence microscope or confocal microscope (Leica TCS SP5, Mannheim, Germany).

### 2.8. RNA Interference (RNAi)

We used the TransIT-X2^®^ Dynamic Delivery System (Mirus, WI, USA) to transfect cells according to the manufacturer’s instructions. CHRNA7 siRNA (ID: NM_000746) was purchased from Sigma Chemical Co. (St. Louis, MO, USA). Briefly, Opti-MEM I reduced-serum medium, siRNA solution and TransIT-X2 were mixed gently. The mixed solution was incubated for 30 min at room temperature to allow the formation of the complexes. Then, the complexes were added to the wells containing the cells for 24–72 h.

### 2.9. Animal Studies

Eight-week-old male C57BL/6 mice (National Laboratory Animal Center, Taipei, Taiwan) were used. The animal protocol was reviewed and approved by the Institutional Animal Care and Use Committee of Taipei Medical University, Taiwan. The mice were randomly separated into three groups (five mice/group): (1) equivalent volumes of saline administered intraperitoneally (i.p.) three times per week for 4 weeks (normal group); (2) mice i.p. injected with 0.5 mg/kg nicotine three times per week for 4 weeks (Nic-0.5 group); and (3) mice i.p. injected with 1 mg/kg nicotine three times per week for 4 weeks (Nic-1 group). At the end of the experimental period, all animals were deeply anesthetized. Blood was collected from the heart, serum separated and stored at −80 °C. They were then killed and sacrificed. Then, the kidney tissues were fixed by formalin and paraffin embedded for histopathological and immunohistochemistry (IHC) staining.

### 2.10. Histological and Immunohistochemical Analysis

The kidneys were fixed in 10% formalin at room temperature for 72 h, then dehydrated and embedded in paraffin. Tissue sections were stained with hematoxylin and eosin (H&E) for histological evaluation. The tubular injury rate of 20 contiguous fields per kidney (5 mice per group) was analyzed. The severity of tubular damage was graded from 0 to 5 according to tubular changes, such as tubular dilatation, flattening of the tubular epithelium and loss of brush borders. The tubular injury score was graded as follows: 0, normal; 1, lesion area <10%; 2, lesion area between 10 and 20%; 3, lesion area between 20 and 30%; 4, lesion area between 30 and 40%; and 5, lesions involving >40% of the field.

For immunohistochemical (IHC) staining, the slides were incubated for 2 h at room temperature with anti-LC3 (MBL, Nagoya, Japan), anti-α7nAChR (Proteintech Group, Chicago, IL, USA), anti-IRE1α (Novus Biologicals, Littleton, CO, USA), anti-KIM-1 (Novus Biologicals, Littleton, CO, USA) or anti-NLRP6 (Bioss Antibodies Inc., Woburn, MA, USA) antibodies. The slides were added with a secondary antibody for 1 h and were displayed using a STARR TREK Universal HRP detection kit (Biocare Medical, Concord, CA, USA). Finally, the slides were stained using hematoxylin.

### 2.11. Detection of Cystatin C by ELISA

The serum of mice was collected to measure cystatin C (Cys C) using ELISA (BioVendor, Brno, Czech Republic) according to the manufacturer’s instructions. The optical density of the peroxidase product was read at 450 nm using an ELISA reader. The concentrations of cystatin C in each sample were determined based on the standard curve.

### 2.12. Statistical Analysis

The results are presented as the means ± standard deviation (SD), and the differences between groups were evaluated using a two-sample t-test or one-way analysis of variance (ANOVA) followed by a post hoc Dunnett’s multiple comparison test. In all statistical tests, *p* < 0.05 was regarded as significant.

## 3. Results

### 3.1. The Cell Viability, ROS Generation and α7nAChR Expression in Human Kidney Cells Treated with Nicotine

**Figure 1:**
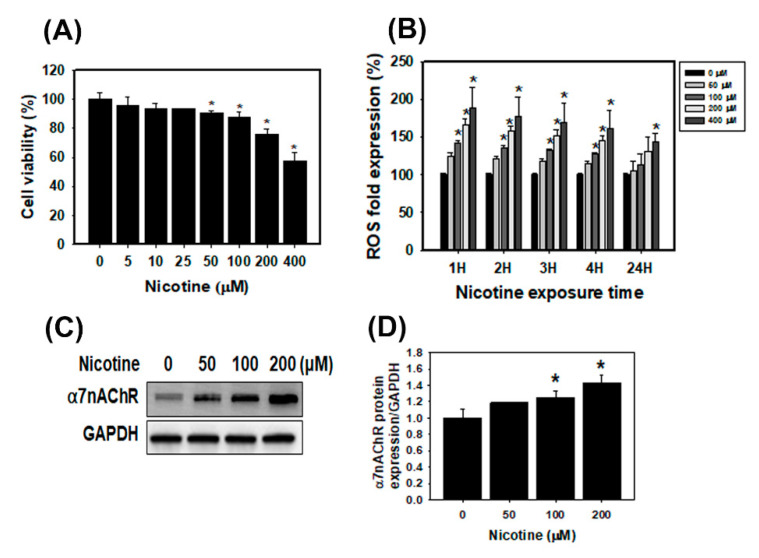
The effects of nicotine on cell viability, ROS generation and α7nAChR expression in human tubular epithelial cells HK-2. (**A**) Cell viability was analyzed using the SRB assay. The HK-2 cells were treated with various concentrations of nicotine for 24 h. * *p* < 0.05 compared with the control. (**B**) ROS generation was assessed using the ROS detection assay kit. The HK-2 cells were treated with various concentrations of nicotine for 1, 2, 3 or 24 h. * *p* < 0.05 compared with the control. (**C**) Western blot analysis was used to assess the expression of the α7nAChR protein in HK-2 cells. The cells were treated with the various concentrations of nicotine for 24 h. (**D**) The α7nAChR protein expression of the histogram represents the average normalized densitometric values. GAPDH was used as the internal control. Data are presented as the means ± standard deviation of three independent experiments. Statistical significance was estimated with ANOVA by Dunnett’s multiple comparison test.

The SRB assay was performed with human kidney proximal tubular epithelial HK-2 cells and rat renal tubular epithelial NRK-52E cells that were treated with nicotine. The viability of HK-2 and NRK-52E cells was observed after treatment with various concentrations of nicotine (0 to 400 μM) for 24 h (Figure 1A and Figure S1A). The result of the SRB assay revealed a decrease in the cell viability of HK-2 and NRK-52E cells exposed to 10–400 μM nicotine. After treatment with 100, 200 and 400 μM nicotine for 24 h, the viability of the HK-2 cells was decreased to 87%, 75% and 57%, respectively. The results of the alamarBlue cell viability assay were also similar to those of the SRB assay (Figure S1B,C). Furthermore, to assess the effect of nicotine exposure on the level of ROS in HK-2 cells, the ROS detection assay, a kit that uses an ROS-sensitive fluorescent dye, was performed. We demonstrated the concentration-dependent and time course effects of nicotine on ROS generation in HK-2 cells (Figure 1B). After nicotine treatment for 1, 2, 3 and 4 h, the intensity of the fluorescence increased in a concentration-dependent manner. In particular, the ROS generation was significantly increased in HK-2 cells that were treated with a high concentration of nicotine (400 μM) for 24 h. Next, we analyzed the HK-2 cells using Western blotting to measure α7nAChR expression. Under the normal condition, no significant α7nAChR expression was detected in the human renal tubular epithelial cells [7]. As shown in Figure 1C, nicotine increased the α7nAChR expression in HK-2 cells. Furthermore, the mRNA levels of CHRNA7 were notably upregulated in HK-2 cells treated with nicotine (Figure S1D). Therefore, nicotine reduced the cell viability, caused the generation of ROS and increased α7nAChR expression in kidney cells.

### 3.2. Nicotine Activates the NLRP6 Inflammasome and Induces ER Stress in Human Kidney Cells

**Figure 2:**
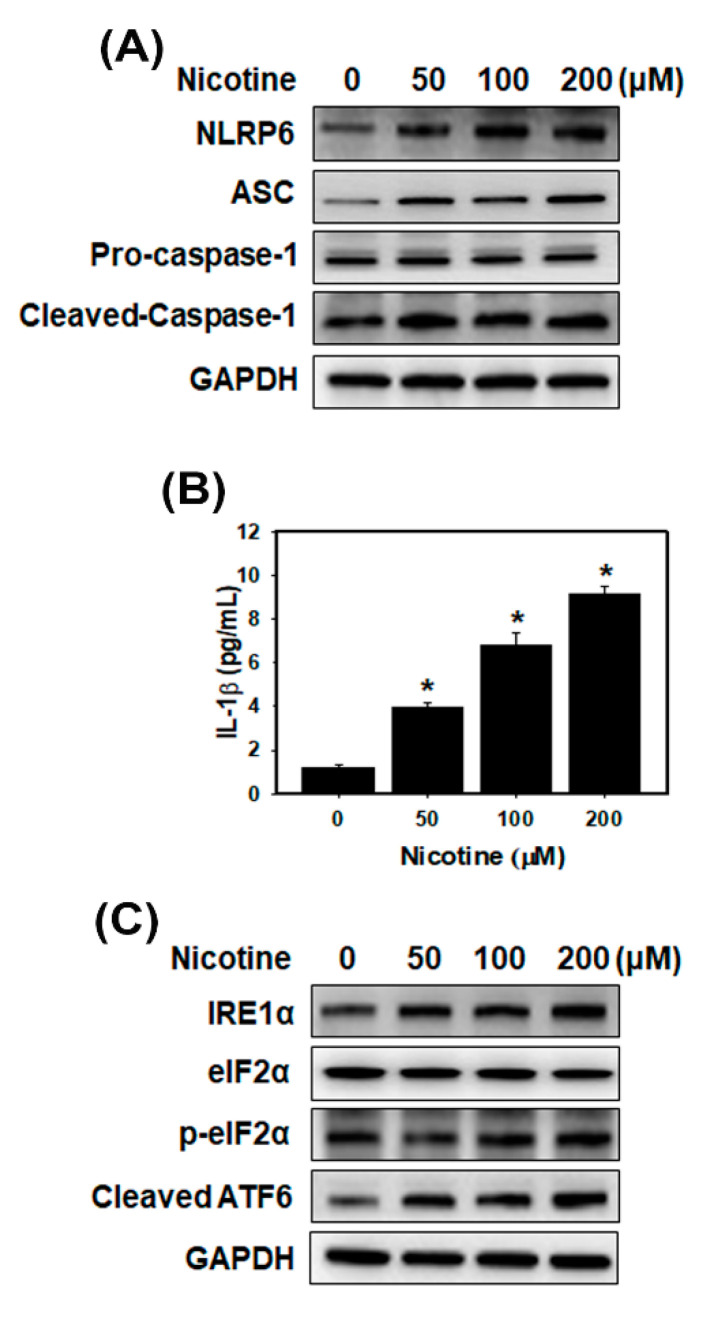
Effects of nicotine treatment on the NLRP6 inflammasome and endoplasmic reticulum (ER) stress in human kidney cells. (**A**) Western blotting for NLRP6 inflammasome-related proteins in HK-2 cells. (**B**) The levels of IL-1β in the culture medium were determined using an ELISA. Data are presented as the means ± standard deviation of three independent experiments. * *p* < 0.05 compared with the control. Statistical significance was estimated with ANOVA by Dunnett’s multiple comparison test. (**C**) Western blotting for ER stress-related proteins in HK-2 cells. The cells were treated with the various concentrations of nicotine for 24 h.

Recent evidence has shown that nicotine causes atherosclerosis via the ROS-NLRP3 inflammasome pathway in endothelial cells [17]. The most studied subunits of inflammasomes are NLRP3 and NLRP6. First, we assessed NLRP3 protein expression using Western blot analysis. However, there was no increase in NLRP3 expression in the HK-2 cells treated with nicotine (Figure S2A). Furthermore, we analyzed the NLRP6 inflammasome-related proteins and a cytokine (IL-1β) (Figure 2A,B and Figure S2B). The results indicated that NLRP6, ASC and cleaved-caspase 1 increased after nicotine treatment in HK-2 cells. Nicotine caused the production of mature IL-1β in a concentration-dependent manner. Therefore, nicotine could induce the NLRP6 inflammasome pathway in kidney cells. A previous study demonstrated that nicotine directly triggered the ER stress response in rat placental trophoblast giant cells and damaged placental function [29]. However, another recent study concluded that nicotine reduced ER stress and improved hepatic steatosis in male rats with diet-induced obesity [30]. Therefore, we evaluated the effects of nicotine on the expression of the ER stress-related proteins in HK-2 cells (Figure 2C and Figure S2C). After incubation of the HK-2 cells with nicotine for 24 h, the cells showed increases in the expression of the IRE1α, p-eIF2α and cleaved ATF6.

### 3.3. Nicotine Induces Mild Apoptosis and Necrosis but Triggers Significant Autophagy in Human Kidney Cells

**Figure 3:**
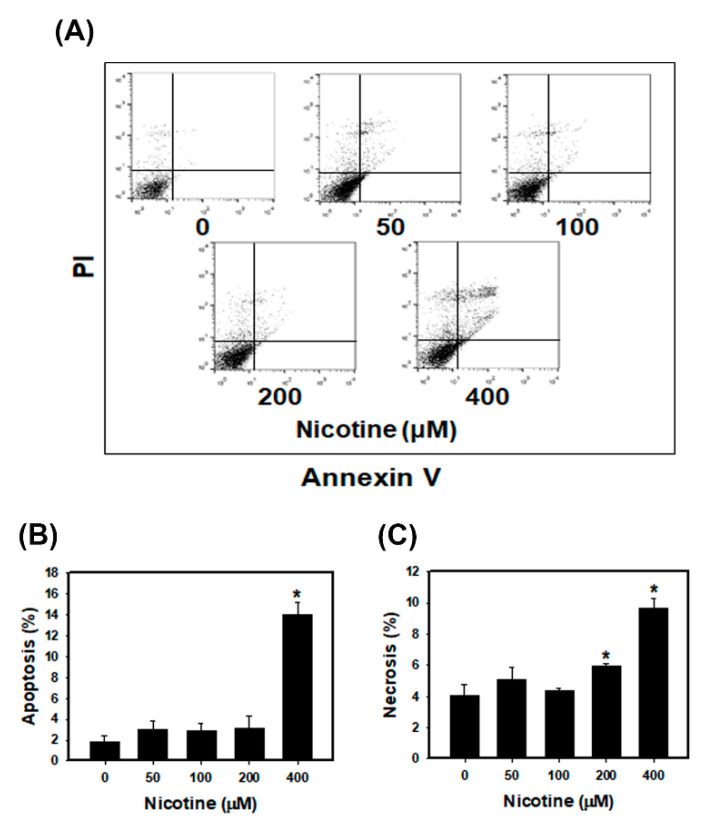
The effects of nicotine on apoptosis and necrosis in HK-2 cells. (**A**) Apoptosis and necrosis were detected using an annexin V/PI staining assay. Quantification of apoptosis (**B**) and necrosis (**C**) in HK-2 cells. The HK-2 cells were treated with various concentrations of nicotine for 24 h. * *p* < 0.05 compared with the control. Data are presented as the means ± standard deviation of three independent experiments. Statistical significance was estimated with ANOVA by Dunnett’s multiple comparison test.

**Figure 4:**
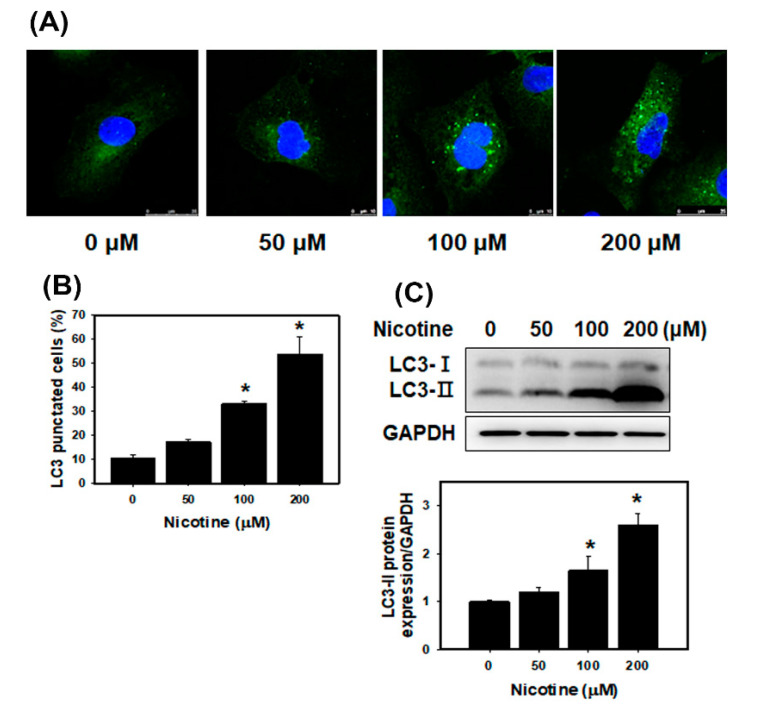
Measurement of autophagy in HK-2 cells that received nicotine treatments. (**A**) Confocal immunofluorescence microscopic imaging of LC3 following 24 h treatment with nicotine. (**B**) Quantification of punctate LC3 staining. * *p* < 0.05 compared with the control. (**C**) The protein levels of LC3 in the HK-2 cells treated with nicotine. The cells were treated with the various concentrations of nicotine for 24 h. The LC3-II protein expression of the histogram represents the average normalized densitometric values. GAPDH was used as the internal control. Data are presented as the means ± standard deviation of three independent experiments. * *p* < 0.05 compared with the control. Statistical significance was estimated with ANOVA by Dunnett’s multiple comparison test.

We assessed the apoptotic and necrotic effects of nicotine using annexin-V binding and PI staining in HK-2 cells (Figure 3). The quantitative results showed that low concentrations of nicotine (50, 100 and 200 μM) did not induce apoptotic or necrotic cell death compared with a control treatment. The percentage of apoptosis in HK-2 cells treated with low concentrations of nicotine was low (less than 4%). Additionally, necrosis was observed in approximately 5.1%, 4.3% and 6% of the cells’ necrosis after treatment with nicotine at 50, 100 and 200 μM, respectively. The HK-2 cells treated with 400 μM nicotine showed remarkable increases in apoptosis and necrosis compared with the control cells. We further examined the effects of nicotine on autophagy in the kidney cells. Previously, the authors reported that ER stress promoted the formation of autophagosomes, and the induction of autophagy can remove toxic misfolded proteins [31]. The accumulated evidence revealed that microtubule-associated protein light chain 3 (LC3) is a marker of autophagy [32]. Therefore, we used fluorescence microscopy to assess the percentage of cells with punctate LC3 staining (Figure 4A,B). The results indicated that nicotine increased the LC3 signals in HK-2 cells in a concentration-dependent manner. Moreover, we analyzed LC3 expression using Western blotting of lysates from HK-2 cells that had been treated with nicotine (Figure 4C). Nicotine treatment increased the expression level of LC3-II.

### 3.4. The Relationship between α7nAChR, NLRP6 Inflammasome and Autophagy in Kidney Cells That Were Treated with Nicotine

**Figure 5:**
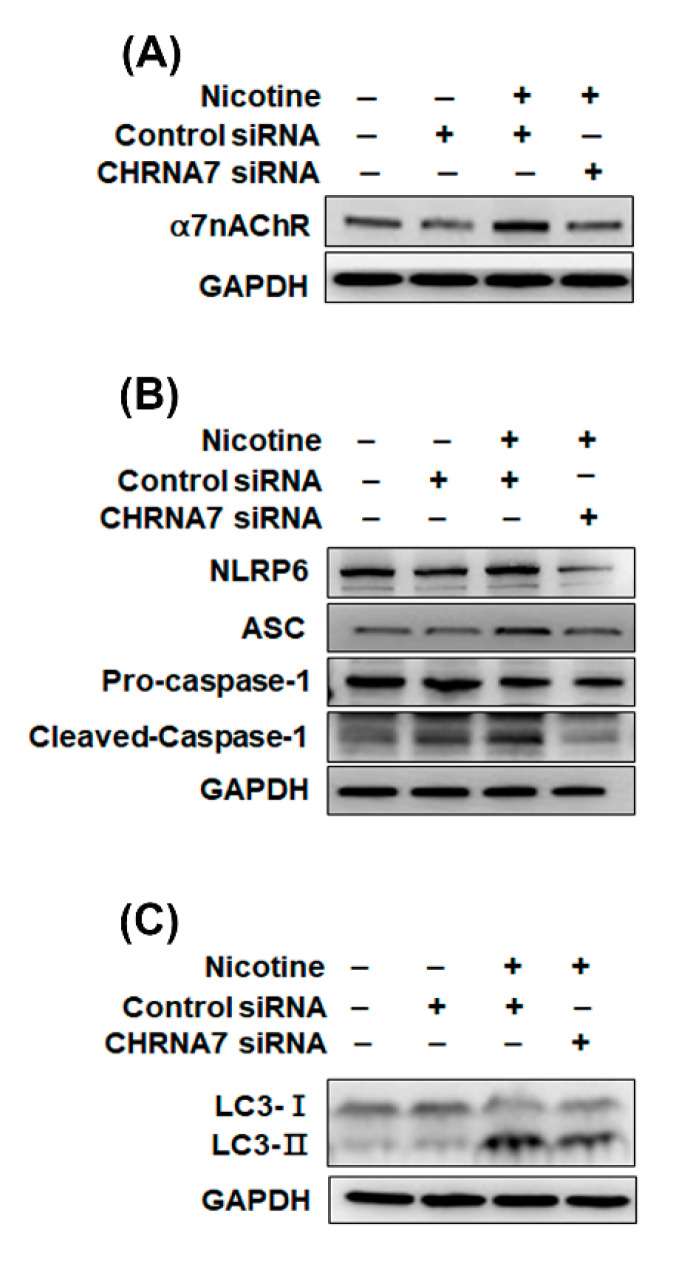
Nicotine induced NLRP6 inflammasomes and autophagy via α7nAChR regulation. (**A**) Western blotting for α7nAChR protein in HK-2 cells treated with control siRNA or CHRNA7 siRNA. (**B**) Western blot analysis of NLRP6 inflammasome-associated protein expression in HK-2 cells. (**C**) The protein levels of LC3 in HK-2 cells. The cells were transfected with control or CHRNA7 siRNA for 24 h and then were treated with nicotine (100 μM) for 24 h. The plus signs indicated to add the nicotine, control siRNA or CHRNA7 siRNA. The minus signs indicated without the nicotine, control siRNA or CHRNA7 siRNA.

As shown in Figure 1C,D, nicotine increased α7nAChR expression in human kidney cells. However, it is currently unclear whether the inflammasomes are influenced by α7nAChR in kidney cells that are treated with nicotine. Recent evidence has shown that α7nAChR can inhibit the NLRP3 inflammasome by regulating β-arrestin-1 in a monocyte/microglia system, thus contributing to the control of neuroinflammation [33]. We analyzed whether α7nAChR could affect the NLRP6 inflammasome. We utilized α7nAChR siRNA to inhibit α7nAChR expression in HK-2 cells. As shown in Figure 5A and Figure S3A, following treatment with nicotine and control siRNA, the α7nAChR expression in HK-2 cells was increased. Transfection with α7nAChR siRNA inhibited the nicotine-induced increases in the α7nAChR protein level. Furthermore, α7nAChR siRNA suppressed the nicotine-induced NLRP6, ASC and cleaved-caspase 1 expression (Figure 5B and Figure S3B). A previous study found that a nonselective nAChR antagonist can reverse nicotine-induced autophagy in vascular smooth muscle cells [22]. However, few published studies have described the relationship between α7nAChR and autophagy. Therefore, we examined whether α7nAChR contributes to autophagy using α7nAChR siRNA. After transfection with α7nAChR siRNA and treatment with nicotine, the effect of nicotine on autophagy was inhibited in HK-2 cells compared with the control siRNA plus nicotine group (Figure 5C and Figure S3C). These results indicated that nicotine induced the NLRP6 inflammasome and autophagy via α7nAChR regulation.

### 3.5. Chronic Nicotine Exposure Results in Renal Injury

**Figure 6:**
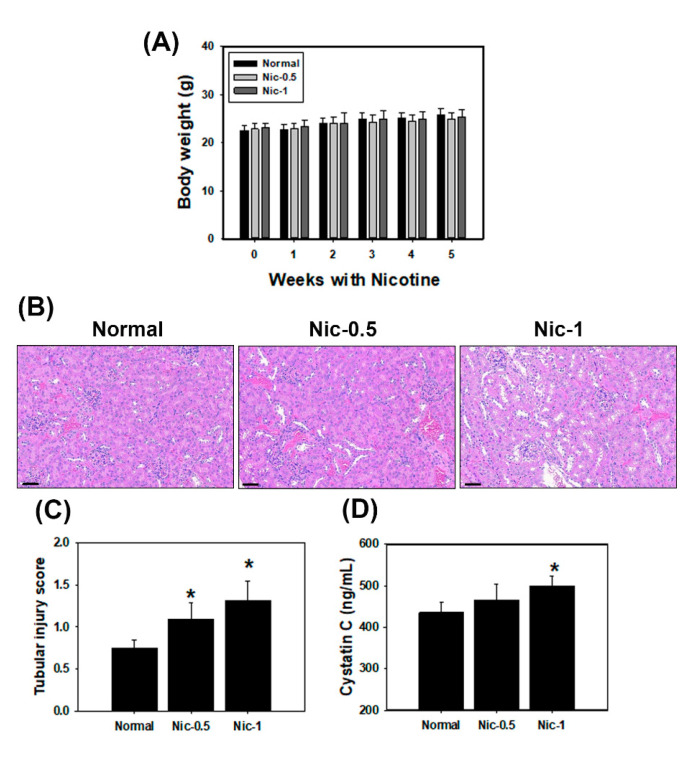
Nicotine increased tubular injury in mouse kidney tissues. Mice were administered with 0.5 and 1 mg/kg nicotine three times per week for 4 weeks. (**A**) Measurement of body weights of C57BL/6 mice in various groups. Data are presented as the means ± standard deviation. (**B**) Representative images of kidney sections in mice were stained with H&E and examined by microscopy. Scale bar = 60 μm. (**C**) The tubular injury score was quantified in kidney sections (5 mice per group). (**D**) The levels of Cys C in the mice serum (5 mice per group) were measured by ELISA. * *p* < 0.05 versus normal group. Data are presented as the means ± standard deviation. Statistical significance was estimated with ANOVA by Dunnett’s multiple comparison test.

**Figure 7:**
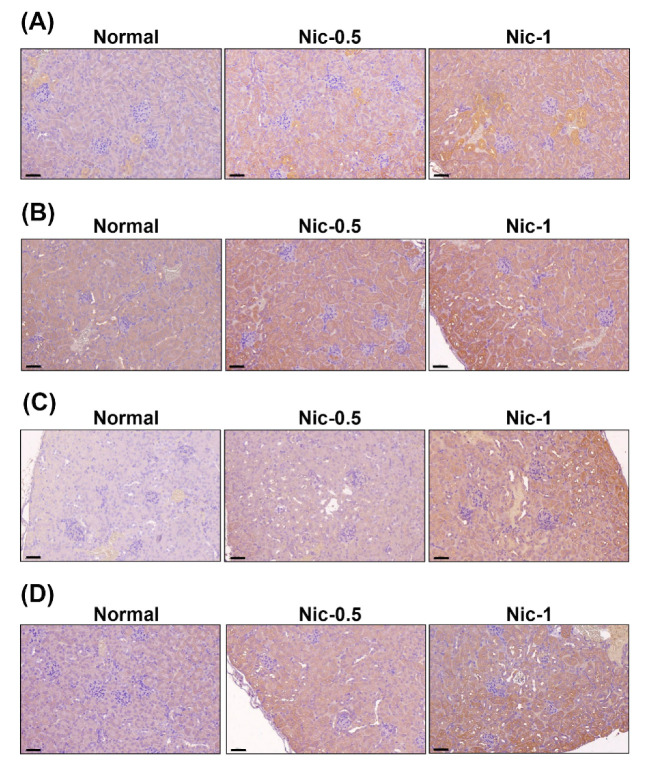
The protein expression of kidneys after nicotine exposure. Immunohistochemistry (IHC) was used to determine the expression levels of α7nAChR (**A**), IRE1α (**B**), LC3 (**C**) and NLRP6 (**D**) in kidney tissues. Scale bar = 60 μm.

To validate the in vitro study, C57BL/6 mice were administrated 0.5 mg/kgand 1 mg/kg nicotine three times per week for four weeks. Our results found that serum creatinine and BUN showed no significance in all nicotine groups compared with the normal group (data not shown). However, the histological analysis in kidney showed evident changes and injury including loss of brush borders, flattening of tubular epithelium and tubular dilatation after treatment with nicotine (Figure 6B). The mean tubular injury scores found in the nicotine group were higher than in the normal group (Figure 6C). The results indicated that nicotine caused tubulointerstitial injury in the in vivo study. Previous research has shown that serum cystatin C was a biomarker of kidney damage and was superior to serum creatinine [34]. An ELISA specific for Cys C was used to quantify cystatin C in mice serum. As shown in Figure 6D, the expression of Cys C in the nicotine groups was higher than that in the normal group. Next, we examined the protein expression by IHC staining in the kidney sections of C57BL/6 mice. It has been reported that kidney injury molecule-1 (KIM-1) is a highly sensitive marker of proximal tubular kidney injury in several animal models [35]. The expression of KIM-1 was significantly increased by nicotine in the kidney tissue (Figure S4). IHC staining revealed that α7nAChR, IRE1α, LC3 and NLRP6 expression was markedly increased in the 0.5 mg/kg and 1 mg/kg nicotine groups compared with the normal group (Figure 7A–D). The results from animal data support our in vitro findings that the NLRP6 inflammasome, autophagy and ER stress play an important role in nicotine-induced kidney damage.

## 4. Discussion

Smoking is an important risk factor and is notable for its ability to exacerbate renal dysfunction [36]. Current cigarette smoking is associated with chronic kidney disease (CKD) or death from end-stage renal disease (ESRD) in both male and female individuals [37]. However, the mechanisms by which cigarette smoking accelerates kidney disease progression have not been identified. Mainstream cigarette smoke involves over 4000 compounds, including carbon monoxide, reactive oxygen species, ketones and stable reactive aldehydes [38]. Among these compounds present in tobacco smoke, nicotine is one of a large number of biologically stable and active compounds present in tobacco that can be obtained through passive and active smoking. Nicotine is responsible for the addictive effects of tobacco smoking and has many biological effects in the pathogenesis of several conditions including kidney diseases [3]. The other chemical substances in tobacco may also cause kidney injury. For example, cadmium (Cd) is present in tobacco smoke and induces damage to the kidneys, the lungs, the bones and the prostate [39,40]. In this study, we focus on the nicotine effect in the kidney. Previous studies have demonstrated that nicotine induced the production of ROS in several epithelial cells including kidney proximal tubular epithelial cells [7,41]. Moreover, nicotine promoted atherosclerosis through ROS-mediated endothelial cell death [17]. In our current study, the observations are similar to those of other investigators. Nicotine caused tubulointerstitial injury (Figure 6B,C and Figure S4). Nicotine activates nAChRs, which function as agonist-regulated Ca2^+^ channels in human mesangial cells [42]. Another study also concluded that nicotine-mediated autophagy accelerates atherosclerosis through the nAChRs/ROS/NF-κB signaling pathway in vascular smooth muscle cells [22]. The accumulated evidence has revealed that α7nAChR plays an important role in the nicotine-promoted progression of renal disease [43,44]. Jain and Jaimes indicated that methyllycaconitine (MLA), which is an α7nAChR blocker, prevents nicotine-induced proteinuria and glomerular injury. Although α7nAChR is predominantly expressed in the proximal tubules, MLA decreases both glomerular injury and interstitial fibronectin expression [3]. Furthermore, α7nAChR induces ROS and activates the PI3K/Akt pathway [45]. Our current findings showed that nicotine significantly increased α7nAChR expression, generated ROS production and reduced cell viability in human kidney cells (Figure 1 and Figure S1). In the in vivo study, nicotine remarkably raised α7nAChR expression in the kidney sections of nicotine groups (Figure 7A). The higher concentrations of nicotine induced apoptosis and necrosis (Figure 3). ROS are pivotal regulators of ER function and UPR activation. It is well known that increased ROS production and ER stress occur together in several diseases [46]. Recent evidence has shown that nicotine directly stimulates the ER stress response in placental trophoblast giant cells and impairs placental function [29]. Another report also found that nicotine exposure during pregnancy damages disulfide bond formation and increases ER stress in the rat placenta [47]. We found that nicotine activated three UPR pathways and ER stress (Figure 2C and Figure 7B).

In this study, we found that nicotine activated the NLRP6 inflammasome and increased IL-1β secretion (Figure 2A,B) but did not induce the NLRP3 inflammasome (Figure S2A) in human kidney tubular epithelial cells. Further, nicotine caused an NLRP6 expression increase in the kidney of the mouse model (Figure 7D). Previous research has shown that α7nAChR inhibited NLRP3 inflammasome activation and prevented the release of mitochondrial DNA which is an NLRP3 ligand. Furthermore, the neurotransmitter acetylcholine suppressed mitochondrial DNA release through α7nAChR and then inhibited NLRP3 inflammasome activation in mouse immune cells [48]. Another recent study concluded that C-reactive protein induced the α7, α9 and α10 nAChRs to inhibit the ATP-mediated monocytic inflammasome [49]. Previous studies have demonstrated that α7nAChR can reduce NLRP3 inflammasome activation by regulating β-arrestin-1 to control neuroinflammation [33]. However, α7nAChR induced ROS [45] and ROS stimulated tissue inflammation through NLRP3 inflammasome activation [50]. Whether α7nAChR has an important role in the nicotine-activated NLRP6 inflammasome remains unknown. In our current study, α7nAChR downregulation suppressed the nicotine-induced NLRP6-related proteins (Figure 5B and Figure S3B). Therefore, nicotine stimulated the NLRP6 inflammasome through α7nAChR.

Autophagy is a unique process that involves intracellular phagocytosis and alterations of the metabolic pathways of eukaryotic cells to maintain cell homeostasis [51]. The level of autophagy in cells is low under normal physiological conditions. However, stress, starvation, infection or ischemia/hypoxia can trigger cellular autophagy, which removes and degrades protein aggregates and damaged organelles to maintain cell viability [52]. The accumulated evidence revealed that autophagy protects kidney cells from injury and apoptosis [53,54]. Gao et al. indicated that nicotine ameliorated colitis by inducing autophagy and had a protective effect against dextran sodium sulfate-induced colitis [55]. Our current findings showed that autophagy was significantly increased in kidney cells that were treated with nicotine (Figure 4 and Figure 7C). Previous research has shown that nicotine induced autophagy in vascular smooth muscle cells (VSMCs) and promoted atherosclerosis through the nAChRs/ROS/NF-κB pathway. Furthermore, the inhibition of nAChRs can reverse VSMC phenotype switching and nicotine-induced autophagy [22]. Methyllycaconitine citrate, which is an α7nAChR blocker, suppresses HO-1 expression and the nicotine-induced autophagy and apoptosis in mouse cardiac myocytes [56]. In this study, we found that nicotine increased autophagy by regulating α7nAChR in human kidney cells (Figure 5C and Figure S3C). However, autophagy can inhibit inflammasome activation [27,28]. Although nicotine induced protective autophagy, nicotine simultaneously triggered other responses such as ER stress and ROS generation. Further, ROS stimulated an inflammatory response and induced inflammasome activation [50].

**Figure 8:**
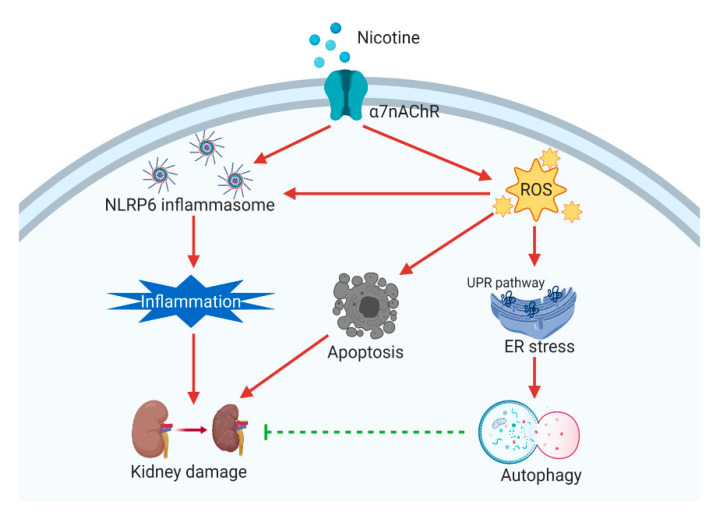
Nicotine causes α7nAChR, NLRP6 inflammasome, ER stress and autophagy in kidney cells. Nicotine induces the NLRP6 inflammasome via α7nAChR. Then, the NLRP6 inflammasome causes inflammation and induces kidney damage. Nicotine triggers autophagy through α7nAChR, and autophagy may protect against nicotine-induced kidney injury. Furthermore, nicotine increases ROS generation and accelerates ER stress by unfolded protein response (UPR) pathways. The high concentration of nicotine can induce apoptosis. Therefore, nicotine causes kidney damage through the modulation of α7nAChR, NLRP6 inflammasome, ER stress and autophagy. The figure was created with BioRender.com.

In conclusion, treatment of kidney cells with nicotine reduced cell viability, generated ROS and increased α7nAChR expression. Moreover, nicotine induced ER stress activation and the NLRP6 inflammasome. Nicotine enhanced mild apoptosis and necrosis but triggered significant autophagy in human kidney cells (Figure 8). In addition, nicotine activated the NLRP6 inflammasome and autophagy through the induction of α7nAChR. In the in vivo study, chronic nicotine exposure resulted in nephrotoxicity. These findings offer us a new understanding of nicotine and the opportunity to find potential preventive or therapeutic strategies for smoking-related renal injury.

## Supplementary Materials

The following are available online at https://www.mdpi.com/2305-6304/8/4/92/s1, Figure S1: The effects of nicotine on cell viability and CHRNA7 mRNA level in tubular epithelial cells. Figure S2: Effects of nicotine treatment on the inflammasome and ER stress in human kidney cells. Figure S3: Nicotine induced NLRP6 inflammasomes and autophagy via α7nAChR regulation. Figure S4: The KIM-1 expression of kidneys after nicotine exposure.
