# Supplementary material for: Nicotine Causes Nephrotoxicity through the Induction of NLRP6 Inflammasome and Alpha7 Nicotinic Acetylcholine Receptor
Source: Toxics. 2020 Oct 26;8(4):92. doi: 10.3390/toxics8040092 (PMC7711477; doi:10.3390/toxics8040092)
Supplement: Supplementary file 1 [file toxics-08-00092-s001.pdf]

toxics

Nicotine causes nephrotoxicity through the induction of NLRP6 inflammasome and alpha7 nicotinic acetylcholine receptor

Cai-Mei Zheng [1,2,3,†], Yu-Hsuan Lee [4], I-Jen Chiu [2,3,5], Yu-Jhe Chiu [2,5], Li-Chin Sung [6,7,8,†] Yung-Ho Hsu [1,2,3,*] and Hui-Wen Chiu [2,3,5,*]

Materials and Methods

AlamarBlue Cell Viability Assay

To seed cells into a 96-well plate containing 100 μL/well of cell culture medium and incubate the cells overnight in a 37°C incubator. After incubation for 24 h with various concentrations of nicotine, the cells were added the alamarBlue cell viability reagent (Thermo Fisher Scientific, Waltham, MA) for 4 h at 37°C in a cell culture incubator. Finally, the optical density was monitored at 570 nm in an ELISA reader.

Reverse Transcription PCR (RT-PCR) and Quantitative PCR (Q-PCR)

Total RNA was extracted from cells using a RNA extraction kit (BIOTOOLS, New Taipei City, Taiwan). Aliquots (5 μg) of total RNA were treated with ToolsQuant II Fast RT kit (BIOTOOLS). TOOLS 2X SYBRTM Green qPCR Mix (BIOTOOLS) was used for Q-PCR by paired primers (CHRNA7: forward-TGGTGACAGTGATCGTGCTGCA and reverse-GCCTCTTCATTCGCAGGAACCA; and GAPDH: forward-CATCACTGCCACCCAGAAGACTG and reverse-ATGCCAGTGAGCTTC CCGTTCAG). The mRNA levels were normalized to those of GAPDH. Fold changes were calculated using the 2−ΔΔCt method.

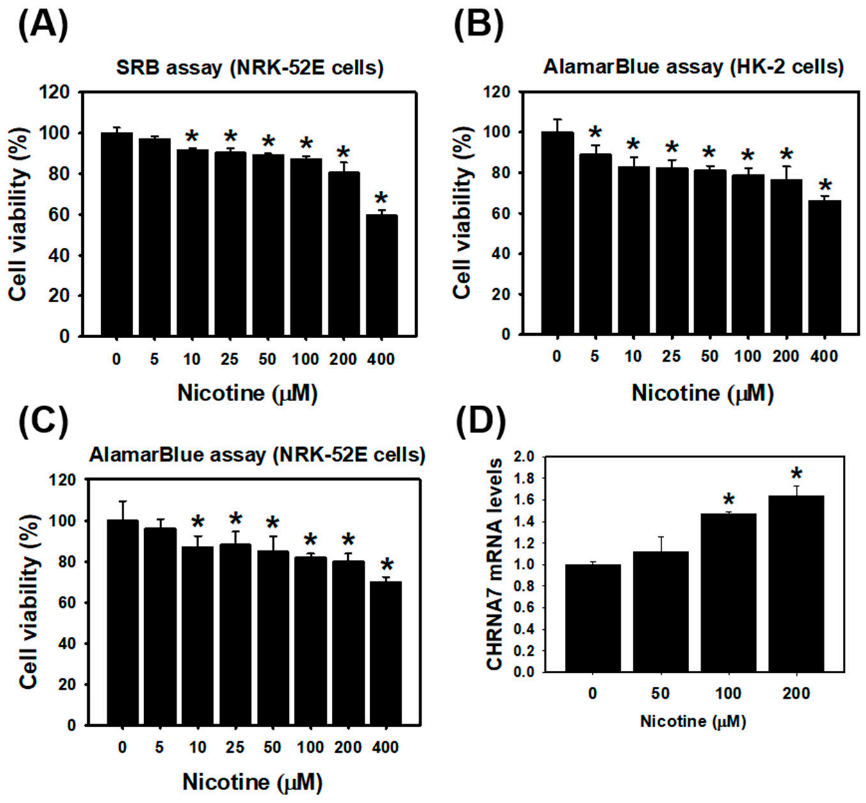

Figure S1. The effects of nicotine on cell viability and *CHRNA7* mRNA level in tubular epithelial cells. (A) Cell viability was analyzed using the SRB assay in NRK-52E cells. The NRK-52E cells were treated with various concentrations of nicotine for 24 h. *P < 0.05 compared with the control. Cell viability was analyzed using the alamarBlue assay in HK-2 (B) and NRK-52E (C) cells. The cells were treated with various concentrations of nicotine for 24 h. *P < 0.05 compared with the control. (D) The mRNA level of CHRNA7 was analyzed in HK-2 cells treated with nicotine for 24 h. *P < 0.05 compared with the control. Data are presented as the means ± standard deviation of three independent experiments. Statistical significance was estimated with ANOVA by Dunnett's multiple comparison test.

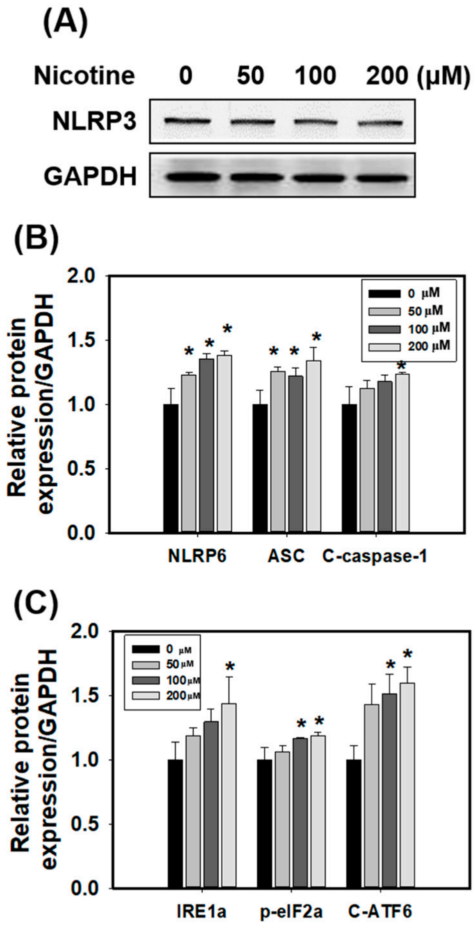

Figure S2. Effects of nicotine treatment on the inflammasome and ER stress in human kidney cells. (A) Western blotting for NLRP3 protein in HK-2 cells. The cells were treated with the various concentrations of nicotine for 24 h. The NLRP6 inflammasome-related proteins (B) and ER stress-related proteins (C) expression of histogram represent the average normalized densitometric values. GAPDH was used as the internal control. Data are presented as the means ± standard deviation of three independent experiments. *P < 0.05 compared with the control. Statistical significance was estimated with ANOVA by Dunnett's multiple comparison test.

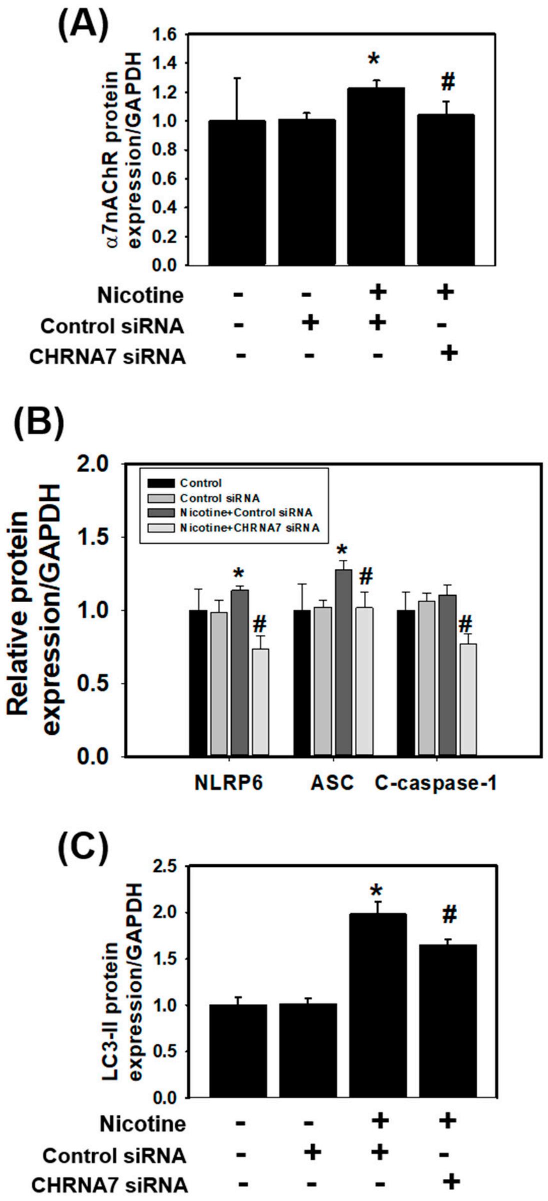

Figure S3. Nicotine induced NLRP6 inflammasomes and autophagy via α7nAChR regulation. (A) The α7nAChR protein expression of histogram represent the average normalized densitometric values. *P < 0.05, nicotine + control siRNA compared with control siRNA. #P < 0.05, nicotine + control siRNA compared with nicotine + CHRNA7 siRNA. (B) The NLRP6 inflammasome-related proteins expression of histogram represent the average normalized densitometric values. *P < 0.05, nicotine + control siRNA compared with control siRNA. #P < 0.05, nicotine + control siRNA compared with nicotine + CHRNA7 siRNA. (C) The LC3-II protein expression of histogram represent the average normalized densitometric values. GAPDH was used as the internal control. The cells were transfected with control or CHRNA7 siRNA for 24 h and then were treated with nicotine (100 μM) for 24 h. Data are presented as the means ± standard deviation of three independent experiments. *P < 0.05, nicotine + control siRNA compared with control siRNA. #P < 0.05, nicotine + control siRNA compared with nicotine + CHRNA7 siRNA. Statistical significance was estimated with *t*-test.

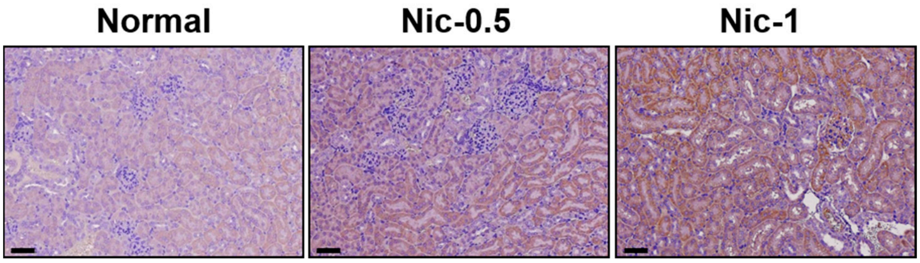

Figure S4. The KIM-1 expression of kidneys after nicotine exposure. IHC was used to determine the expression levels of KIM-1 in kidney tissues. Scale bar=60 μm.
